# The Lived Experience of Pain Services: A Comparison of Service Users' and Service Providers' Experience of Irish Health Services

**DOI:** 10.1155/prm/4608906

**Published:** 2025-08-03

**Authors:** Kate Sheridan, Aine MacNamara, Enda Whyte, Siobhan O'Connor

**Affiliations:** School of Health and Human Performance, Dublin City University, Dublin, Ireland

**Keywords:** chronic pain, healthcare support, lived experience, self-management

## Abstract

**Background:** A supportive healthcare experience that implements a biopsychosocial model of care can empower a person with chronic pain to make informed decisions and engage in self-management behaviours. Despite the positive influence of supportive healthcare, little is known about the presence of healthcare support in under-resourced chronic pain services. This idiographic study explores the lived experience of service users and providers participating in chronic pain services with a specific focus on autonomy support and self-management skills.

**Methods:** Semistructured interviews were conducted on service users (*n* = 7) self-reporting a diagnosis of chronic pain (pain > 3 months) and service providers (*n* = 5), defined as healthcare professionals with > 3 years of experience in clinical healthcare settings managing pain conditions. All interviews took place online (mean 47 ± 11 min). Interview transcripts were analysed using interpretative phenomenological analysis.

**Results:** Analyses generated four themes: ‘biomedical model leads care'; ‘lost in a system'; ‘I need support' and ‘the essentials of self-management'. Both service users and providers described regular experiences of invalidation and biomedical approaches to pain management. Long waitlists, a lack of multidisciplinary services, short appointment times and a lack of educational resources all impacted the development of self-management skills in service users.

**Conclusion:** Despite clinical guidelines recommending a biopsychosocial model of care, the biomedical model remains the dominant approach in chronic pain management, reflecting a persistent gap between evidence and practice. Service users and providers desire access to multidisciplinary services that support a biopsychosocial model of care. Healthcare professionals cannot deliver what service users expect due to macro-, meso- and microlevel factors. Future research is needed to explore practical solutions to deliver pain services that optimise the development of self-management skills where existing infrastructure and resources negatively impact service delivery. Suggested approaches include enhancing autonomy-supportive communication by healthcare providers and ensuring early access to high-quality educational materials.

## 1. Introduction

Chronic pain is a life changing experience for those who live with it [1]. Affecting one in three people in Ireland [2], chronic pain impacts multiple aspects of a person's life including, their psychological health, occupational performance, personal relationships and life satisfaction [1, 3]. Chronic pain requires multidimensional treatments to reduce burden and optimise quality of life [4, 5]. Therefore, a biopsychosocial model of care addressing the dynamic interaction of physiological, psychological and social factors associated with chronic pain is warranted [6, 7]. A biopsychosocial approach can be delivered clinically through the implementation of patient-centered care [8]. Patient-centered care ensures patients are informed, empowered, share in the decision-making process, and develop self-management skills in a partnership with their healthcare professional (HCP) [9–11].

An interdisciplinary pain management program (PMP) is recognised as the gold standard intervention to provide patient-centered care and introduce self-management skills [12]. In Ireland, just five multidisciplinary PMPs are available nationally within the public health system [13]. In the absence of PMPs, often due to limited resources, individual clinicians are tasked with providing patient-centered care and self-management strategies for living with pain. Autonomy-supportive healthcare is a core pillar of providing patient-centered care [14]. According to self-determination theory (SDT), autonomous behaviour is dependent on three basic psychological needs: autonomy, competence, and relatedness [15]. Autonomy supportive healthcare environments are settings where HCPs fulfil the three basic psychological needs of SDT, thereby supporting patients to engage in autonomous health-related behaviours and self-management practices [16]. Autonomy supportive healthcare results in positive changes to both psychological and physical health [14, 17]. While SDT provides a useful framework for understanding patient motivation, meaningful engagement in self-management also requires attention to external factors, including access to services, social support and health system infrastructure. Therefore, both motivational and structural supports must be considered when evaluating or developing chronic pain services.

There is a dearth of research exploring the lived experience of pain services in Ireland. Research from a decade ago reports long wait lists, frustration with the healthcare system and challenges in navigating fragmented services [18]. Furthermore, the limited availability of PMPs in healthcare services is a potential limitation to the delivery of patient-centered care for chronic pain sufferers. Internationally, there is an acknowledged gap between current evidence and clinical practice in pain management, which may affect the implementation of pain care [19]. These findings, combined with the high prevalence of chronic pain in Ireland, raise concerns about the lived experiences of both people currently living with pain and HCP providing services nationwide. Therefore, this study aimed to explore the lived experience of service users seeking care for chronic pain and service providers participating in Irish healthcare services, specifically focusing on autonomy support and self-management behaviours.

## 2. Methods

### 2.1. Design

Interpretative phenomenological analysis (IPA) was chosen due to its idiographic nature, providing a detailed exploration of the participants' personal lived experience [20]. IPA is a valuable methodology in chronic pain/healthcare research as it explores complex biopsychosocial phenomena [21, 22].

### 2.2. Participants

Persons living with chronic pain (service users) (*n* = 7) were purposefully recruited from a medical exercise clinic in Ireland. Inclusion criteria were: (1) aged between 18 and 65 years, (2) experiencing chronic pain for ≥ 3 years and (3) having received pain management care from ≥ 3 different service providers. Individuals unable to participate in an English-language interview were excluded. These criteria were chosen to ensure participants had diverse and sufficient clinical experiences to reflect on. Service providers (*n* = 5) with ≥ 3 years working with persons living with chronic pain were recruited via a targeted email invitation and snowball sampling. In this study, the term service provider refers to HCP involved in the management of individuals living with chronic pain, including general practitioners, consultants, allied health professionals, and nurses. As promoted by IPA, a detailed account of individual experiences and interpretation of these lived experiences, rather than thematic saturation, was the focus of the study, and a sample size of 12 individuals was considered satisfactory [23]. Participants were given up to 4 weeks to review the study information and return the signed informed consent form prior to data collection. To ensure anonymity, service users were not direct patients of service providers accepted for the study. Ethical approval was granted by the DCU Research Ethics Committee (DCUREC/2023/039).

### 2.3. Procedure

Individual, semistructured interviews were completed on the online Zoom platform (Zoom Video Communications Inc., San Jose, California) offering logistical benefits to both researchers and participants [24]. All interviews were conducted by the first author who has a 12 year history of working as a Certified Athletic Therapist and Chartered Physiotherapist specialising in pain management and an experienced clinical interviewer. At the time of recruitment, the first author had transitioned from a previous clinical role at the medical exercise clinic and returned solely in a research capacity. While some service users were familiar with the researcher due to this prior clinical role, the author was not involved in their care preceding the study. The service providers were recruited from separate facilities. While the first author was familiar with some through professional networks, there were no direct working relationships or collaborations prior to the study. Each interview commenced with the question “Tell me about your experience with chronic pain services in Ireland?” Next, exploratory prompts and questions, informed by SDT [25], were used to encourage elaboration and provide participants with the opportunity to discuss topics pertinent to them. As such the interview guide for service users (Supporting Information S1) and the interview guide for service providers (Supporting Information S2) were used in a way that allowed participants to lead the interview and for new topics to be explored as they arose. As well as audio recordings, the interviewer made written notes during the interviews to include nonverbal communication, new topics introduced by the participant and personal reflections on the interview process. Interviews ceased when participants confirmed they had exhausted their personal accounts. Interviews ranged from 32 to 63 min (mean 47 ± 11 min). A practice interview was conducted prior to recruitment to review interview technique and clarity of questions. No changes were made to the interview guide following this review.

### 2.4. Data Analysis

Data analysis was completed as per the framework of analysis detailed by Smith and Fieldsend [20] described in Table 1.

### 2.5. Reflexivity and Trustworthiness

Throughout data collection the first author was aware of the importance of considering her own precognitions and influence on participants and data interpretations. Bracketing was employed to allow reflection on the data and consideration of how any subjectivity interacted with the data analysis [26]. One of the co-authors acted as a critical friend, challenging the meaning units developed by the first author [27] through conversations focused on ‘How might my background be influencing how I interpret this experience' and ‘Am I over-interpreting based on my own preconceptions'. The resulting themes were discussed and agreed upon by a team of three co-authors, an interdisciplinary group comprising Chartered Physiotherapists, Certified Athletic Therapists and qualitative researchers. Finally, given the importance of presenting the lived experience of the participants, thick description is used through significant direct quotations to preserve the richness of the participant's accounts. Given the importance of credibility and trust, the steps taken to develop a level of rapport with participants were important [28]. Credibility and rapport were also enhanced by the first author's positioning in a professional role and her clinical experience in the area, which may have increased the likelihood of participant openness.

## 3. Results

Service users (*n* = 7) included 4 females and 3 males, had a median age of 57 years (range 36–65), and experienced pain for a median of 30 years (range 3–40) (Table 2). Service providers (*n* = 5) included 4 females and 1 male, had a median age of 46 years (range 38–60) and had a median of 12 years of clinical experience in practice (range 8–30) (Table 3). None of the service users had been referred to a multidisciplinary PMP to engage in a self-management program by their service provider. Six participants were unaware that self-management programs existed, with one participant being advised their knowledge exceeded what a self-management program could offer. Only one of the five service providers had access to refer patients to a PMP which had a 2 year waiting list. Final analysis of the data, incorporating perspectives from both service users and service providers, highlighted four group experiential themes, Biomedical Model Leads Care, Lost in a System, I Need Support, and The Essentials of Self-Management, as summarized in Table 4. Each theme includes subthemes, which are illustrated with verbatim quotes.

### 3.1. Biomedical Model Leads Care

This theme referred to participants' accounts of how the biomedical model of care dominated their experience and comprised of two subthemes: ‘Medication led management', and ‘A desire for biopsychosocial model of care'.

#### 3.1.1. Medication Led Management

All service users and three of the five service providers reported pharmacology as a first-line treatment for chronic pain. Despite the limitations of pharmacology in treating certain chronic pain conditions, such as chronic low back pain, some service providers felt pressure to employ a medication-led approach with one GP acknowledging “*it's probably the easiest thing to do but probably the least effective method of dealing with chronic pain”.* All service providers and service users reported a negative experience when pharmacology was not effective in treating chronic pain, often experienced at the individual level. For the service user this was described as *“I was made to feel guilty that I was failing the medication rather than the medication wasn't working for me” (Service User 4).* While for the service providers there was a sense that *“you'll reach the end of the road (prescribing) with those patients, and you feel that there's nothing left for them” (Neurologist)*. Although all service providers vocalised the importance of also adopting nonpharmacological therapies in pain treatment, service users universally reported a resistance by service providers to believe that nonpharmacological therapies such as modifications to lifestyle behaviours could have a positive impact on pain.

“*When I started seeing an improvement and I was suggesting that it was me moving to a holistic approach, he didn't believe me and thought it might have been the medication that I was on, but I had been on that medication for like 3 years at the time and nothing had happened until I started the different holistic things” (Service User 4)*

#### 3.1.2. A Desire for Biopsychosocial Model of Care

All service users expressed a desire for care that was individualised and addressed all aspects of their personal health needs. This desire for a patient centered model of care was exemplified by one service user who expressed their frustration at not having their values, experiences or personal context considered in their care plan suggesting a homogenous approach to care, “*I'm not sure if I was 20 years older if the treatment would be much different” (Service User 6)*. All service users expressed a clear desire for support that extended beyond their physical symptoms to include both psychological and social aspects of health. For example, one service user described the loneliness associated with chronic pain and the lack of available support to address this, while another recalled scoring poorly on a psychological outcome measure collected by their consultant but receiving no follow-up support to manage this clinically significant finding: “*I scored pretty low, I needed support from a psychological point of view*” (*Service User 1*). Service users described the suffering associated with receiving a diagnosis without any additional social or psychological care: “*I was told by my neurologist that I probably wouldn't walk or work again and was then sent off to deal with that information without any support*” (*Service User 4*).

Despite a predominantly medication-led management approach, service providers all acknowledged the importance of psychological and social support in pain management. A gap in psychological implementation was evident with four service providers highlighting the limited availability of psychologically trained professionals in their service and three service providers expressing concern relating to their professional capacity to support psychological wellbeing with the physiotherapist noting; *“you just don't know where the line is crossed within the threshold of my professional scope of practice*”. In this regard, all service users and service providers noted the need for a multidisciplinary team with expertise in pain management to support the implementation of a biopsychosocial model of care. The lack of provision was highlighted as a significant factor with MDT care not available in the private sector unless service users self-referred and individually paid for each service. Despite a reported presence of MDTs in the public sector there was agreement by service providers that they do not ‘*exist in a meaningful way*' due to limited staffing and long waitlists.

### 3.2. Lost in a System

This theme ‘Lost in a system' refers to the ambiguous experiences reported in navigating pain services and comprises two subthemes: ‘No clear pathway' and ‘Accessibility'.

#### 3.2.1. No Clear Pathway

All service users and four service providers described an unclear treatment pathway for chronic pain. For example, one service user, described that *“I just didn't know where to go, what to do, who to believe”* (*Service User 5*). Interestingly, the metaphor of fog was utilised by both a service user and a service provider when speaking about treatment pathways, illustrating the confusion, uncertainty and unknowns present in relation to pain services. The physiotherapist described how she associated the lack of clarity of pain pathways to a treatment “*merry-go-round that the patients are constantly on*”.

Although one service user viewed their GP as the gatekeeper to coordinate their pain care, all other service users expressed a desire for an individual service provider who showed leadership and direction in their pain care. One service user described how *“there's no overall sort of helicopter view of your problem”* (*Service User 2*). There was agreement between service providers that the available treatment pathways do not provide optimal standards of pain care, and that there was motivation for change despite ongoing resource limitations with the recognition that services had improved significantly in the last 10 years *“We are not really there … But we are moving in the right direction for sure”* (Pain Consultant).

#### 3.2.2. Accessibility

Delayed access to MDT services due to waitlists and under-staffed services was noted by all service providers as a blockade to essential pain care. The GP expressed their frustration with access to services noting that “*we have no access to psychology and physio is similarly bad. Allied health professionals are much better at chronic pain than we are, but it's unfortunately left to us to deal with*”. Speed of access was reported as highly important by all service providers in the challenge of treating chronic pain. The neurologist, rheumatologist and pain consultant all described their services as being overwhelmed by the demands placed upon them and this impacted their ability to review patients in a timely manner. These accessibility issues, with participants reporting wait times of between 1 and 4 years, had significant impacts on the service user.

Increased support staff and new pain treatment algorithms were all proposed as solutions to more timely access to pain care. However, despite the best efforts of service providers, delays in recruitment and available funding affected their efforts to enhance service provision. This failure to expand services was illustrated by the Pain Consultant who spent 5 years applying for funding for an advanced nurse practitioner to support their pain service before approval. Furthermore, all service users and service providers noted that access to pain treatment was only available in hospital settings reflecting a frustration with the lack of community-based resources, *“The HSE (Health Service Executive) has a responsibility to provide better access to resources within the community and trying to get these things out of the hospital”* (GP).

All service users and four service providers expressed how the length of current appointment times *“10 min is not going to get you through an explanation”* (GP) affected the therapeutic relationship and was insufficient for meaningful conversations to support long-term engagement in self-management strategies. Simultaneously, service users sympathised with the pressures on service providers to ‘*churn through*' a high number of appointments daily; *“For a consultant you probably have a 15 min window to try and explain what has been going on for potentially 6 month and dancing all that into 15 min slot it's just, it's not doable”* (*Service User 4*). In contrast, enhanced satisfaction was reported where service users spent 30 min or more in appointments.

### 3.3. I Need Support

This theme highlights the fundamental requirement of support, the need for encouragement, empathy and assistance, desired by both service users and providers in navigating pain care. It is comprised of two subthemes ‘I can't do it alone' and ‘pain invalidation'.

#### 3.3.1. I Can't Do It Alone

Reflecting a perception that they felt isolated, both service users and service providers reported a need for support to successfully navigate and implement pain services. The level of support desired by service users varied and was influenced by the frequency, severity, and nature of pain, including its changing quality and the emotional distress it caused. For example, there was agreement across service users that they could largely support themselves when pain levels were low but during flares of pain, they desired increased support from service providers. For one participant the metaphor of a ‘rabbit hole' was used to describe becoming overwhelmed and caught in the complexities of their pain experience, emphasising their need for external support to regain perspective and emerge from a dark place.

*“You need someone to take a look and say, okay, hang on here now you're struggling too much because you don't realize how far down that rabbit hole you're going” (Service User 7)*

Support was seen by service users as a balance between being encouraged and pushed outside of their comfort zone. When service users felt well supported, they described that experience as ‘life changing'. Feeling supported was associated with being given hope for the future and feeling cared for but was not associated with resolution of pain symptoms. Despite the desire for support, most service users noted that their support needs were not met; *“You're on your own. Definitely. I would love to be in a room when someone said, that's not the case. I would challenge that heavily”* (*Service User 1*). Interestingly, three service users expressed a desire for pain support services to have the same visibility and access as cancer support services. They described a desire to have their hand held, to be provided with high levels of information, and to feel supported like other long-term conditions.

*“With the cancer support services, it's there, where it really isn't there for pain you're given all this information and your hand held and you know, you're very well looked after, really supported environment, very supportive environment.”* (*Service User 7*)

Service providers also expressed a feeling of isolation and a need for enhanced support for themselves to continue to provide pain care. Service providers described the experience of working in pain care as stressful, challenging and mentally exhausting. A number of service providers noted their distress at feeling inadequate in the service they provide, the physiotherapist described; *“I don't know if we are doing anything really well to be honest. I definitely think support for health professionals is needed”*. Furthermore, two of the service providers expressed hopelessness that a number of colleagues would shift from a biomedical model of care to considering the biopsychosocial needs of the person in pain noting ‘*it's probably a waste of resources trying to make them*'. These reflections highlight both the systemic and peer support needs of service providers working in chronic pain care.

#### 3.3.2. Pain Invalidation

All service users and three service providers reported experiencing invalidation of pain within health services. Service users described being told the pain was ‘in their head' and ‘feeling like hypochondriacs'. One service user reported his disappointment at feeling dismissed and misunderstood; “*Half of them [healthcare professionals] don't want to know and half of them [healthcare professionals] don't seem to understand*” (*Service User 6*). Both the physiotherapist and rheumatologist also expressed concern at ongoing invalidation of chronic pain by their colleagues; *“there are always going to be people [clinicians] that don't believe it, won't accept it, won't acknowledge it”* (Rheumatologist). Both service providers recalled the negative impact they witnessed on their service users when they interacted with service providers who invalidated the complexities and challenges associated with living with an invisible illness.

Service users reported that validation from service providers was heavily reliant on visible pain behaviours and expressions of low psychological wellbeing. One service user recalls the need to perform ‘*an act*' in order to receive adequate validation of their pain; *“You want to go in there and really look like you're in a very, very poor state, and they might do something. But if you walk in there, dressed, showered and upright, you're ticking the boxes.”* Two service providers expressed surprise at the positive impact that resulted from talking with service users. The neurologist noted *“sometimes you spend an hour with them in clinic not doing anything just listening to them”.* These communication skills such as active listening were perceived as an important step for service users to ‘*feel believed*'. In contrast, many service users described how they were not listened to, or consulted, as exemplified by service user 4: *“he doesn't listen. He'll just fire questions until he gets some sort of an answer that he wants.”* This experience of not feeling heard had a negative influence on service user experience.

### 3.4. The Essentials to Self-Management

This theme highlighted the value that both service users and providers placed on self-management strategies in managing chronic pain. Developing self-management skills was associated with three subthemes ‘Exercising autonomy', ‘Expanding knowledge' and ‘Supported behaviour change techniques'.

#### 3.4.1. Exercising Autonomy

Service users all agreed that their experience in health services improved when they developed the confidence to express their opinions and contribute to the decision-making process of their pain management. One service user described autonomy as *“trying to work out what would be the right solution for me as opposed to the clinician saying this is the right thing for you”.* Although four service providers described supporting service user autonomy, all service users had experienced paternalistic interactions where they did not feel supported to exercise autonomy by service providers with one service user recalling *“it was very difficult to kind of shift that relationship so that you are taking more control”*. Service users expressed distress, regret and frustration at the lost time where they perceived they had no control or input to their treatment plan with one service user expressing; “*Maybe I should have pushed a little bit more maybe gone somewhere else, you know I just took what he said was wrong with me and did what he said.” (Service User 5).* While the physiotherapist acknowledged the healthcare system can make it “*difficult for them [service user] to find their voice*” all other service providers noted the need for service users to take the lead, be proactive and demonstrate internal motivation in order to self-manage their care. Despite the desire for service user proactivity, one service user noted the significant challenge of exercising autonomy while consumed with high levels of pain, *“basically the legs had been kicked out from under me. So, I was just kind of going on along with whatever treatment plan.” (Service User 4).*

Service users associated autonomy support with feeling cared for by their service providers. Feeling cared for was dependent on the time taken to explain a diagnosis, the empathetic nature of the professional, their relatability, kindness, and honesty. Interestingly, service users did not report that feeling cared for was associated with the service providers level of experience or ability to resolve the pain.

*“He just cared about my quality of life, and he really wanted to in so far as possible, to have me back out there. And yeah, he showed that he cared, and he put himself out there to try and do anything that he could within his capacity.” (Service User 1)*

Service users reported feeling a lack of care and understanding where the chronic pain condition was the focus of the clinical interactions. One service user identifies the dehumanising experience of being considered by their diagnosis and not as a whole person; *“I think they could read the lines on paper, but they just they didn't get what does that mean?” (Service User 1).*

#### 3.4.2. Expanding Knowledge

All participants expressed a desire for additional educational resources and pathways of care to enhance knowledge. All service providers reported the importance of pain education in clinical appointments and their priority in increasing service user's knowledge. Only the physiotherapist and GP reported that time constraints may affect the success of education in current clinical practice describing their ability to deliver “*a snippet of education*” which by their reflection did not meet the needs of the person in pain. Despite service providers acknowledging the importance of knowledge, all service users had independently accessed patient advocacy support groups online to support their knowledge of pain management and were frustrated at not being guided to these free resources by service providers. Service users attributed a lack of knowledge upon receiving a pain diagnosis as the primary reason that delayed them in exercising autonomy and developing self-management skills. One service user described the problem of ‘*unknown unknowns*' expressing the significant challenge of advocating for oneself when there are significant gaps in your knowledge while another service user noted *“it takes a lot to try and find those resources when you're so low on energy”.* A lack of time to source trustworthy resources, uncertainty about which materials were evidence-based, and limited access to free tools prevented service providers from confidently signposting service users to appropriate educational supports.

#### 3.4.3. Supported Behaviour Change Techniques

Three service providers reported frustration at not having the ability ‘*to fix*' chronic pain and therefore recognised the need to engage the service user in health behaviours that support self-management. All service providers admitted that despite initial guidance and early-stage education they could not provide long-term assistance nor were community resources available to support behaviour change techniques that would assist living with pain. Service users expressed a desire to be able to self-manage their symptoms and improve their quality of life and also highlighted the lack of available supports in the community to develop the required skills. Despite the joint agreement on the need for self-management a number of service users felt dismissed by their service providers and their efforts to engage in what they considered to be health behaviour changes such as lifestyle adaptions, conservative and holistic therapies. Service users described the process of developing self-management skills as an isolating experience that was primarily self-guided and involved a series of trial and errors. Despite the frustration at navigating the behaviour change techniques without the support of healthcare services, for many service users the eventual act of developing self-management skills was empowering, confidence building and provided a significant sense of achievement. For one service user, developing self-management skills was initially challenging and unsupported. However, over time, through personal experimentation and perseverance with lifestyle-related strategies, such as tapering off medication, adjusting daily routines (e.g., pacing and exercise) and using mental health techniques, they began to feel more in control of their condition. This process of self-discovery led to a strong sense of personal ownership and capability, as reflected in their account: *“I feel more capable of managing it now than I ever have and the improvements that I've seen since coming off medication and taking charge of the ownership of managing it has been huge.”* (Service User 4).

## 4. Discussion

This study aimed to explore the lived experience of service users and providers participating in Irish healthcare services specifically focusing on autonomy support and self-management behaviours. Participants in this study described their experiences of pain services centered strongly on a biomedical care model where the primary focus is pain severity reduction using pharmacology and interventional pain medicine. This is despite evidence that improvements in pain and disability are recorded where pharmacological and interventional treatments are delivered in combination with physical, psychological, and behavioural therapies [29]. Although service providers acknowledged the need for nonpharmacological therapies, long waitlists, staff resources and a lack of multidisciplinary referral pathways obstructed a biopsychosocial model of care. Significantly, none of the service users in this study were referred to a PMP, and only one service provider reported having direct access to refer patients to a PMP. While all service providers were aware of PMPs, these findings highlight both limited referral access and gaps in knowledge or utilisation of PMPs within the Irish healthcare context.

It is of concern that both service users and providers experienced invalidation of pain, particularly where symptoms were not visible. The pain invalidation observed in this study corresponds with evidence that an absence of visible pain results in service users feeling stigmatised and receiving less empathy from HCPs [30, 31]. This finding of pain invalidation is surprising given the widespread knowledge that pain is a subjective experience and that a person's report of pain should be respected, regardless of physical signs [32]. This is particularly important in chronic pain, where individuals may learn to mask symptoms or suppress behaviours typically associated with pain in order to appear socially acceptable and avoid disrupting social norms [33]. Interestingly, the desire for chronic pain to be approached with the same support as cancer services has been recorded previously and highlights the desire for increased visibility and acceptance of chronic pain as a diagnosis [34].

Importantly, service users in this study expressed a desire for autonomy, competence in self-managing pain, and a preference for feeling cared for as a whole person, aligning with the three basic psychological needs of SDT (autonomy, competence, and relatedness) [35]. Despite their reported desire to be autonomous when making decisions about their pain care, service users found that high pain levels, a lack of confidence and vulnerability while living with pain affected their ability to act autonomously, particularly early in their diagnosis. Service users expressed a need for significant support in achieving autonomy and competence in their care. The barriers to autonomy supportive healthcare that service users reported including controlling language, judgement of choices and limited appointment times have been reported previously [36]. In comparison service providers agreed that appointment times affected service provision but also reported accessibility to allied health professionals and a lack of referral pathways in the community as further barriers. Importantly, insufficient time in appointments has been reported as a direct barrier to implementing the biopsychosocial approach [37].

Neither an early introduction to self-management skills nor direction to evidence informed resources were reported by service users despite service providers acknowledging the importance of education and resources. Service users described learning to advocate for themselves over time by self-educating and conducting their own research [38]. Self-management development was therefore driven by service user's dissatisfaction with pain services rather than a treatment practice encouraged by service providers. Service users require support and shared decision-making practices to develop health behaviours, learn problem solving skills and engage in activities led by their individual values and goals [39]. Prior research advises that HCPs have admitted their need for additional skills in supporting self-management to address the challenge of chronic pain [40]. The need for enhanced service provider support is reflected in our findings that service providers reported concern for their own wellbeing as well as concern of extending outside of their professional scope of practice in supporting service user's psychological health.

Ultimately the results of this study echo the pain management evidence-practice gap highlighted internationally [19]. This study's results align with research that highlights the continued dominance of the biomedical model in pain care settings [38, 41–43]. The dominance of the biomedical model for chronic pain management is in direct contrast to clinical guidelines calling for a biopsychosocial model of care [44–46]. The findings of this study echo previous research that a review of processes at macro- (healthcare policy), meso- (funding, resources) and microlevels (HCP factors) of health services is essential in order to deliver the desired biopsychosocial model [37]. Future research should focus on identifying effective, scalable strategies to support the development of self-management skills within the constraints of existing healthcare infrastructure. One promising opportunity to provide early educational resources for those living with chronic conditions is eHealth strategies [47]. Additionally, research should evaluate the impact of autonomy-supportive communication and validation training for healthcare providers, as well as explore system-level interventions aimed at reducing delays and resource limitations in Irish pain services.

There are a number of limitations to the above study. Service users who had long histories of chronic pain treatment and service providers with experience in numerous healthcare settings were chosen for this study in an attempt to explore a rich variety of lived experiences of pain in Ireland; however service users were not direct patients of service providers accepted for the study impacting the comparison of experiences. Furthermore, service providers taking part in this study primarily worked in private health care services at the time of data collection, however it is noted that all service providers had completed medical training in public health settings and could detail experiences in both environments.

## 5. Conclusion

The lived experience of pain by both service users and providers highlights a conflict between a desire for autonomy supportive environments that support self-management skills and the available resources to provide such experiences. An individual service provider alone cannot resolve the concerns highlighted in this study; rather to support service providers a review of performance is needed at macro-, meso- and microlevels. At a microlevel service providers can improve the pain experience of service users by active listening, enforcing pain validation, supporting autonomy and providing educational resources promoting self-management early in clinical interactions. Further research is required to identify where pragmatic changes, such as the integration of eHealth tools, implementation of autonomy-supportive communication strategies, and system-level interventions to improve resource allocation, can be applied to enhance the experiences of both service users and service providers within Irish pain services.

## Figures and Tables

**Table 1:** IPA data analysis [20].

IPA data analysis	Action
Step 1 familiarise with transcript	Transcribe interviews verbatim. Read and reread transcribed interview while listening to audio recording
Step 2 exploratory noting in right-hand margin	Adopt a phenomenological focus by exploring semantic content and language
Step 3 experiential statements in left-hand margin	A synergistic process of combining the analyst's interpretation and the participant's lived experience
Step 4 development of personal experiential statements (PETs)	Conceptual ordering of experiential statements to form personal experiential statements
Step 5	Repeat steps 1–4 for each participant
Step 6 development of group experiential themes (GETs)	Explore convergence and divergence across all participants PETs to create group experiential themes (GETs)

**Table 2:** Service users demographics.

Service user	Sex	Age (years)	Years of pain	Pathology	HCP
1	Male	45	3	Cauda equina	GP, PT, OT, rehabilitation consultant
2	Male	65	30	Chronic low back pain	GP, PT, pain consultant, consultant neurosurgeon
3	Female	57	40	Degenerative spine disease and ankylosing spondylitis	GP, PT, orthopaedic consultant, consultant neurosurgeon, pain consultants, rheumatologist
4	Female	36	8	Chronic migraine	GP, neurologist, consultant in internal medicine
5	Female	59	30	Chronic low back pain, rheumatoid arthritis,	GP, PT, rheumatologist
6	Male	45	11	Chronic neuropathic pain	GP, PT, consultant neurosurgeon, pain consultant
7	Female	63	40	Rheumatoid arthritis, psoriatic arthritis & chronic low back pain	GP, PT, orthopaedic consultant, pain management consultant, rheumatologist

Abbreviations: GP = general practitioner, HCP = healthcare professionals engaged with since chronic pain diagnosis, OT = occupational therapist, PT = physiotherapist.

**Table 3:** Service providers demographics.

Service provider	Sex	Age	Years in practice	Public/Private	Access to refer to PMP	Wait list for PMP
GP	Female	60	30	Both	No	
Neurologist	Female	38	8	Both	Yes	2 years
Pain consultant	Male	51	12	Private	No	
Rheumatologist	Female	46	15	Private	No	
Physiotherapist	Female	36	11	Private	No	

Abbreviations: MDT = multidisciplinary team, PMP = pain management program.

**Table 4:** Group experiential themes.

Group experiential themes	Subthemes
1. Biomedical model leads care	i. Medication led management
	ii. A desire for biopsychosocial model of care

2. Lost in a system	i. No clear pathway
	ii. Accessibility

3. I need support	i. I can't do it alone
	ii. Pain invalidation

4. The essentials of self-management	i. Exercising autonomy
	ii. Expanding knowledge
	iii. Supported behaviour change techniques

## Data Availability

The data that support the findings of this study are available on request from the corresponding author. The data are not publicly available due to privacy or ethical restrictions.

## Supporting Information

Supporting Information 1 Supporting Information S1: Interview guide for service users.

Supporting Information 2 Supporting Information S2: Interview guide for service providers.
