# Supplementary material for: The Lived Experience of Pain Services: A Comparison of Service Users' and Service Providers' Experience of Irish Health Services
Source: Pain Res Manag. 2025 Aug 3;2025:4608906. doi: 10.1155/prm/4608906 (PMC12335910; doi:10.1155/prm/4608906)
Supplement: Supporting Information 1 — Supporting Information S1: Interview guide for service users. [file 4608906.f1.docx]

**Supplementary Materials S1 – Service User Interview Guide**

**Introduction and definitions**

*Research suggests that supportive healthcare environments enhance pain rehabilitation. That is, informed options and support relating to available therapies and lifestyle changes could enhance your quality of life. The purpose of this interview is to help us understand more about the barriers and facilitators to support in chronic pain services.*

*I’m going to ask you a few questions about your perspective on the support and therapy options you received from your healthcare professional. Please describe as much of your experience as possible. If there is a question that you would prefer not to answer or you need a break, that is no problem and it will not affect your relationship with me or any organisation that you may be associated with.*

*Before we start do you have any questions for me?*

*Are you happy to start the interview?*

| Definition | | |
| --- | --- | --- |
| Autonomy Supportive Healthcare Environment | Autonomy supportive environments support a person’s autonomy (feeling free to engage in a behaviour), competence (feeling effective to engage in a behaviour) and relatedness (feeling cared for and valued). | Ntoumanis et al. (2020)  Williams et al. (1998) |
| Barriers | Factors that make it harder for your healthcare professional to provide autonomy support | Fuller et al., (2019) |
| Facilitators | Factors that make it easier for your healthcare provider to provide autonomy support | Fuller et al., (2019) |

**Demographic Details: Service User**

1. What is your age (in years)? ______

2. What is your gender? *Circle below*

1. Male
2. Female
3. Non-binary
4. Other
5. Prefer not to say

4. How long have you had your pain? _____years _____ months

5. What is the cause of your chronic pain? ___________________________

6. Who do you consider your primary Healthcare Professional?* _______________

(E.g GP, consultant, nurse, physiotherapist etc)

*Your Primary Healthcare Professional is the person you consider has the most input and influence on your chronic pain treatment.

7. Do you see them in the public or private Healthcare system? __________________

8. Have you been on a Pain Management Program? _______________

| Question | Prompt | Rationale |
| --- | --- | --- |
| Opening Question:  Tell me about your experience with chronic pain services in Ireland? | Who is your primary health professional?  How long have you seen them? | Opening Question, Ice Breaker |
| Do you feel that your healthcare professional has provided you with choices and options to treat your chronic pain? | Can you describe the choices you were given to treat your condition?  You mentioned __________ were any further treatments discussed? | Autonomy Support / shared decision making  Biomedical Healthcare versus Biopsychosocial care |
| Do you feel your healthcare professional has made sure you really understand about your condition and what you need to do to reduce your pain and optimise your rehabilitation? | Can you give an example of how they did/ didn’t do this?  What do you understand is happening with your condition? | Autonomy Support / shared decision making |
| Do you feel that your healthcare professional cares about you as a person? Why? | Does your healthcare professional listen to how you would like to do things?  Do you think your healthcare professional considers your emotions? | Relatedness |
| Are you able to be open with your healthcare professional during your appointments? Why/Why not? | What things do you feel you can’t express in your appointments? | Autonomy support/ value led rehabilitation |
|  |  |  |
| In your opinion what are the main barriers to you receiving (autonomy) support from your healthcare professional? | How do you think these barriers can be overcome?  What is missing from the pain service you received? | Shared decision making / patient centred care |
| What are the main facilitators to you receiving autonomy support from your healthcare professional? |  |  |
|  |  |  |
| Do you feel motivated to manage your chronic pain? | Are you doing it for yourself (internally motivated) or doing it for other people (externally motivated) | Internal versus external motivation |
| Do you feel competent to manage your chronic pain? | 1. What other factors influence your competence? 2. What does competence mean to you? | Competence / valued care / self-management |
| What does competence mean to you? |  | Competence / valued care / self-management |
| Do you feel competent to engage in physical activity? |  | Competence / valued care / self-management |
|  |  |  |
| Do you feel like you have a shared partnership with your healthcare professional in deciding the best treatment and lifestyle changes for your pain? | Are your values and lifestyle considered when a treatment plan is formulated? |  |
