# Supplementary material for: The Lived Experience of Pain Services: A Comparison of Service Users' and Service Providers' Experience of Irish Health Services
Source: Pain Res Manag. 2025 Aug 3;2025:4608906. doi: 10.1155/prm/4608906 (PMC12335910; doi:10.1155/prm/4608906)
Supplement: Supporting Information 2 — Supporting Information S2: Interview guide for service providers. [file 4608906.f2.docx]

**Supplementary Materials S2 – Service Provider Interview Guide**

**Introduction and definitions**

*Research suggests that supportive healthcare environments enhance pain rehabilitation. The purpose of this interview is to help us understand more about the barriers and facilitators to supporting chronic pain service users.*

*I’m going to ask you a few questions about your perspective on the support and therapy options you offer as a healthcare professional. Please describe as much of your experience as possible. If there is a question that you would prefer not to answer or you need a break, that is no problem and it will not affect your relationship with me or any organisation that you may be associated with.*

*Before we start do you have any questions for me?*

*Are you happy to start the interview?*

| Definition | | |
| --- | --- | --- |
| Autonomy Supportive Healthcare Environment | Autonomy supportive environments support a person’s autonomy (feeling free to engage in a behaviour), competence (feeling effective to engage in a behaviour) and relatedness (feeling cared for and valued). | Ntoumanis et al. (2020)  Williams et al. (1998) |
| Barriers | Factors that make it harder for your healthcare professional to provide autonomy support | Fuller et al., (2019) |
| Facilitators | Factors that make it easier for your healthcare provider to provide autonomy support | Fuller et al., (2019) |

| Question | Prompt | Rationale |
| --- | --- | --- |
| Opening Question:  Tell me about your experience working in chronic pain services? | What type of patients do you often see with chronic pain? What experiences have they had before they get to your clinic? Does that make your role harder?  How challenging is it to manage chronic pain in your clinic? | Opening Question, Ice Breaker |
| Do you feel that in your clinic you are able to provide chronic pain patients with choices and options to treat their chronic pain? | As a GP/ consultant what choices can you offer to patients with chronic pain?  You mentioned __________ are there any further treatments available that your clinic can’t offer? Why? | Autonomy Support / shared decision making  Biomedical Healthcare versus Biopsychosocial care |
| In your clinic do you feel that patients understand their chronic pain condition and what role they can take to reduce their pain and optimise their rehabilitation?  Any patient advocacy groups you recommend?  What resources do you think would support chronic pain patients being able to take control of their pain?  Are these resources available in your clinic? | Can you give an example of how they did/ didn’t do this? | Autonomy Support / shared decision making |
| In your clinic are you able to talk to your patient about the emotional or psychological impact of their pain? Why, why not?  Is there an available pathway for you to refer patients for psychological/emotional support?  How important is psychological support in chronic pain? | Do you listen to how your patient would like to do things? Such as their treatment preferences | Relatedness |
| Do you think patients think they can be open with you during your appointments? Why/Why not? |  | Autonomy support/ value led rehabilitation |
|  |  |  |
| In your opinion what are the main barriers to you providing the support required to your patient when they are managing a chronic pain condition?  Is their a toll on you working with long-term pain? | How do you think these barriers can be overcome?  What is missing from the pain service we provide? | Shared decision making / patient centred care |
| What are the main facilitators to you providing chronic pain support/ treatment?  What is working well in your clinic to provide a good service for these patients? |  |  |
|  |  |  |
| Do you feel patients with chronic pain are motivated to manage their chronic pain? | Are you doing it for yourself (internally motivated) or doing it for other people (externally motivated) | Internal versus external motivation |
| Do you think in Ireland we support people to become competent to manage their chronic pain? | 1. What factors influence a person to develop competence? 2. What does competence mean to you? | Competence / valued care / self-management |
| What does competence mean to you? |  | Competence / valued care / self-management |
| Do you think patients with chronic pain are competent to engage in physical activity?  Do you have resources/ support available to assist you to support chronic pain patients to engage in physical activity? | What services are missing to allow this to happen?  Do you have pathways you can advice patients to follow to get support with this? | Competence / valued care / self-management |
|  |  |  |
| Do you feel like you are able to offer a shared partnership with your patients where you both decide the best treatment and lifestyle changes for their pain? | Are their values and lifestyle considered when a treatment plan is formulated? |  |

What do we need to do next for chronic pain services?

**Demographic Details: Service Provider**

1. What is your age (in years)? ______

2. What is your gender? *Circle below*

1. Male
2. Female
3. Non-binary
4. Other
5. Prefer not to say

4. How long have you worked in chronic pain services? _____years _____ months

5. What clinic do you work in? ___________________________

7. Do you see them in the public or private Healthcare system? __________________

8. Have you referred patients onto an MDT based Pain Management Program? _______________ (Do you have access to PMP?)
